# A Fast Feature Selection Algorithm by Accelerating Computation of Fuzzy Rough Set-Based Information Entropy

**DOI:** 10.3390/e20100788

**Published:** 2018-10-13

**Authors:** Xiao Zhang, Xia Liu, Yanyan Yang

**Affiliations:** 1 Department of Applied Mathematics, School of Sciences, Xi’an University of Technology, Xi’an 710048, China; 2 Department of Automation, Tsinghua University, Beijing 100084, China

**Keywords:** information entropy, fuzzy rough set theory, feature selection, fast algorithm

## Abstract

The information entropy developed by Shannon is an effective measure of uncertainty in data, and the rough set theory is a useful tool of computer applications to deal with vagueness and uncertainty data circumstances. At present, the information entropy has been extensively applied in the rough set theory, and different information entropy models have also been proposed in rough sets. In this paper, based on the existing feature selection method by using a fuzzy rough set-based information entropy, a corresponding fast algorithm is provided to achieve efficient implementation, in which the fuzzy rough set-based information entropy taking as the evaluation measure for selecting features is computed by an improved mechanism with lower complexity. The essence of the acceleration algorithm is to use iterative reduced instances to compute the lambda-conditional entropy. Numerical experiments are further conducted to show the performance of the proposed fast algorithm, and the results demonstrate that the algorithm acquires the same feature subset to its original counterpart, but with significantly less time.

## 1. Introduction

Rough set theory [1] presented by Pawlak in 1982 is a useful tool to deal with vagueness and uncertainty information in the field of computer sciences. The research of rough set theory has mainly focused on both the generalizations of rough set models and the applications in different data environments, which has already attached much attention in granular computing [2,3,4], feature selection [5,6,7,8], dynamic data mining [9,10,11], and big data mining [12,13]. On the other hand, since the information entropy is powerful to measure information uncertainty, it has been extensively applied in practical problems, such as decision making [14], time series [15], portfolio selection [16], and so on.

In view of the effectiveness of information entropy to measure uncertainty in formation, information entropy has been extensively applied in the rough set theory to mine knowledge, which mainly concentrates on constructing rough set-based entropy in different information systems to measure the significance of features (or attributes) or the quality of knowledge granules and on exploring practical applications of rough set-based entropy. Specifically, in the aspect of constructing rough set-based entropy [17,18,19,20,21,22,23,24,25,26,27,28], the references [18] and [19] respectively introduced the concepts of information entropy, rough entropy, and knowledge granulation in complete and incomplete information systems and provided their important properties. Hu et al. [20] proposed the generalizations of the entropy to calculate the information of a fuzzy approximation space and a fuzzy probabilistic approximation space, respectively. Xu et al. [21] introduced the definition of rough entropy of rough sets in ordered information systems. Mi et al. [22] formulated the entropy of the generalized fuzzy approximation space. Dai and Tian [25] provided the concepts of knowledge information entropy and knowledge rough entropy in set-valued information systems, and investigated their properties. Dai et al. [26] presented the rough decision entropy to evaluate the uncertainty of interval-valued decision systems. Chen et al. [27] introduced the neighborhood entropy to evaluate the uncertainty of neighborhood information systems. Wang et al. [28] put forward a unified form of uncertainty measures for general binary relations.

In the aspect of exploring practical applications of rough set-based entropy [29,30,31,32,33,34,35], Pal et al. [31] defined the measure “rough entropy of image” for image object extraction in the framework of rough sets. Tsai et al. [32] provided an entropy-based fuzzy rough classification approach to acquire classification rules. Chen and Wang [33] presented an improved clustering algorithm based on both rough set theory and entropy theory. Sen and Pal [34] gave classes of entropy measures based on rough set theory to quantify the grayness and spatial ambiguity in images. Chen et al. [35] put forward an entropy-based gene selection method based on the neighborhood rough set model. Furthermore, it is worth noting that one of the most important applications of rough set-based entropy is feature selection (attribute reduction) [36,37,38,39,40,41,42,43,44]. For example, Miao and Hu [36] defined the significance of attributes from the viewpoint of information and then proposed a heuristic attribute reduction algorithm by using the mutual information. Wang et al. [37] developed two novel heuristic attribute reduction algorithms based on the conditional information entropy. Hu et al. [39] introduced a fuzzy entropy to measure the uncertainty in kernel approximation based on fuzzy rough sets, and thus proposed the feature evaluation index and a feature selection algorithm. Sun et al. [40] provided the rough entropy-based uncertainty measures for feature selection in incomplete decision systems. Liang et al. [41] introduced the incremental mechanisms for three representative information entropies and then developed a group incremental entropy-based feature selection algorithm based on the rough set theory with multiple instances being added to a decision system. Chen et al. [43] proposed a neighborhood entropy to select feature subset based on the neighborhood rough set model. Zhang et al. [44] presented a feature selection method by using the fuzzy rough set-based information entropy.

Since the computation of the fuzzy rough set-based information entropy in [44] is quite time-consuming, we propose in this paper a corresponding improved mechanism with lower complexity to compute the entropy and develop a fast feature selection algorithm that can quickly obtain the same result to the feature selection algorithm in [44]. In addition, the performance of the fast algorithm is shown by some numerical experiment.

In the remainder of this paper, we briefly review in Section 2 the feature selection algorithm in [44] and some related knowledge. In Section 3, the computational properties of the fuzzy rough set-based information entropy in [44] are presented. A fast feature selection approach with lower complexity has been developed. Numerical experiments were documented in Section 4 to show the performance of the proposed fast feature selection algorithm.

## 2. Preliminaries

As indicated in [45], a fuzzy information system is a pair (U,A) in which $U=\{x_{1},\;x_{2},\;\ldots ,\;x_{n}\}$ is the universe of discourse and $A=\{a_{1},\;a_{2},\;\ldots ,\;a_{m}\}$ is the attribute set. For each attribute $a_{t}\in A$, a mapping $a_{t}:U\to V_{a_{t}}$ holds where $V_{a_{t}}$ is the domain of $a_{t}$, and a fuzzy relation $R_{\left\{a_{t}\right\}}$ can be defined. The fuzzy relation of a subset B⊆A is $R_{B}=\underset{a_{t}\in B}{⋂}R_{\left\{a_{t}\right\}}$.

It is possible to define the corresponding fuzzy relations for the attributes with different types of values, and one can refer to [44] for the details. Here, a fuzzy relation *R* is a fuzzy set that is defined on the fuzzy power set F(U×U) to measure the similarity between two objects in the universe *U*.

By adding an attribute set D={d} with A∩D=∅ into a fuzzy information system (U,A), we obtain a fuzzy decision system (U,A∪D) where *A* is the conditional attribute set and *D* is the decision attribute set. It should be pointed out that *d* is a nominal attribute on which a mapping $d:U\to V_{d}$ holds and $V_{d}$ is the domain of *d*.

By utilizing a fuzzy rough sets-based information entropy, a forward addition feature selection algorithm is proposed in [44], and it is as follows.

**Algorithm 1:** Computing an ε-approximate reduct of a fuzzy decision system.

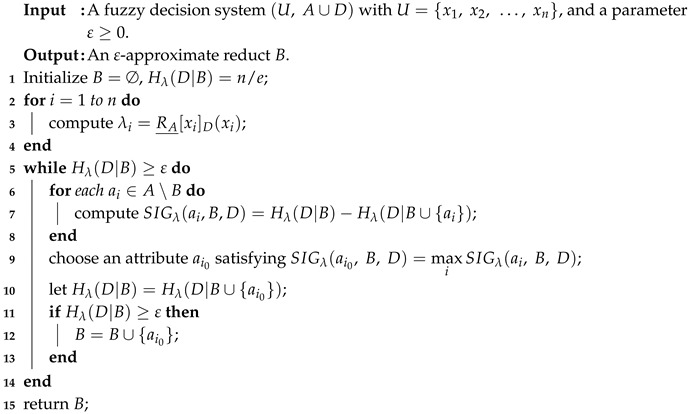



In Step 3 of Algorithm 1, $\underline{R_{A}}{\left[x_{i}\right]}_{D}$ is the fuzzy lower approximation of the decision class ${\left[x_{i}\right]}_{D}$ based on the fuzzy relation $R_{A}$, which is proposed in the pioneering work of fuzzy approximation operators [46] and is concretely computed by

(1) $$\underline{R_{A}}{\left[x_{i}\right]}_{D}\left(x_{i}\right)=\underset{x_{j}\in U}{\inf}\max\left\{1-R_{A}\left(x_{i},\;x_{j}\right),\;{\left[x_{i}\right]}_{D}\left(x_{j}\right)\right\}.$$

Here, ${\left[x_{i}\right]}_{D}$ is the crisp decision class to which the object $x_{i}$ belongs, and ${\left[x_{i}\right]}_{D}=\left\{x_{j}\in U:\left(x_{i},\;x_{j}\right)\in R_{D}\right\}$ where $R_{D}$ is the equivalence relation generated by the nominal decision attribute *d*. Thus, the membership function of the decision class ${\left[x_{i}\right]}_{D}$ is

(2) $${\left[x_{i}\right]}_{D}\left(x_{j}\right)=\left\{\begin{array}{c} 1,\;x_{j}\in {\left[x_{i}\right]}_{D};\\ 0,\;\mathrm{otherwise} \end{array}.\right.$$

In Step 7, $SIG_{\lambda }\left(a_{i},\;B,\;D\right)$ is the significance of the attribute $a_{i}$ ($a_{i}\in A\setminus B$) for *B* relative to *D*, which is factually the decrease of the λ-conditional entropy in the process of adding one attribute. Here, the λ-conditional entropy of the decision attribute set *D* relative to the conditional attribute subset *B*, i.e., $H_{\lambda }\left(D|B\right)$, is defined in [44] as

(3) $$H_{\lambda }\left(D|B\right)=-\frac{1}{n}\sum_{i=1}^{n}\left(\left|{\left[x_{i}\right]}_{B}^{\lambda_{i}}\cap {\left[x_{i}\right]}_{D}\right|\log\frac{\left|{\left[x_{i}\right]}_{B}^{\lambda_{i}}\cap {\left[x_{i}\right]}_{D}\right|}{\left|{\left[x_{i}\right]}_{B}^{\lambda_{i}}\right|}\right)$$

where

(4) $${\left[x_{i}\right]}_{B}^{\lambda_{i}}\left(x_{j}\right)=\left\{\begin{array}{c} \lambda_{i},\;1-R_{B}\left(x_{i},x_{j}\right)<\lambda_{i};\\ 0,\;\mathrm{otherwise} \end{array}\right.$$

is the fuzzy granule of $x_{i}$ with respect to *B*, and $\lambda_{i}=\underline{R_{A}}{\left[x_{i}\right]}_{D}\left(x_{i}\right)$.

It should be pointed out that |X| is the cardinality of the fuzzy set *X*, which is defined in [38] as $\left|X\right|=\sum_{i=1}^{n}X\left(x_{i}\right)$. For example, $\left|{\left[x_{i}\right]}_{B}^{\lambda_{i}}\right|=\sum_{i=1}^{n}{\left[x_{i}\right]}_{B}^{\lambda_{i}}\left(x_{j}\right)$. Moreover, as indicated in [44], if there exists an object $x_{i_{0}}\in U$ such that $\lambda_{i_{0}}=\underline{R_{A}}{\left[x_{i_{0}}\right]}_{D}\left(x_{i_{0}}\right)=0$, then take

(5) $$\left|{\left[x_{i_{0}}\right]}_{B}^{0}\cap {\left[x_{i_{0}}\right]}_{D}\right|\log\frac{\left|{\left[x_{i_{0}}\right]}_{B}^{0}\cap {\left[x_{i_{0}}\right]}_{D}\right|}{\left|{\left[x_{i_{0}}\right]}_{B}^{0}\right|}=0.$$

Generally, the λ-conditional entropy is less than n/e. Thus, the λ-conditional entropy $H_{\lambda }\left(D|B\right)$ is initialized to n/e in Step 1 of Algorithm 1. Furthermore, the λ-conditional entropy is of monotonicity, i.e., $H_{\lambda }\left(D|C\right)\geq H_{\lambda }\left(D|B\right)$ holds for C⊆B⊆A, which yields $SIG_{\lambda }\left(a_{i},\;B,\;D\right)\geq 0$. Therefore, in the iteration procedure of Algorithm 1, the feature $a_{i_{0}}$ satisfying $SIG_{\lambda }\left(a_{i_{0}},\;B,\;D\right)=\max_{i}SIG_{\lambda }\left(a_{i},\;B,\;D\right)$ is added in a feature subset.

As indicated in [44], the time complexity of Algorithm 1 is $O(|U{|}^{2}|A{|}^{2})$, in which Step 7 is the critical step to select features and the complexity of computing $SIG_{\lambda }\left(a_{i},\;B,\;D\right)$ is $O(|U{|}^{2})$, as well as the complexity of running Steps 2–4 is $O(|U{|}^{2}|A|)$. Here, |·| is the cardinality of one crisp set. Computing $SIG_{\lambda }\left(a_{i},\;B,\;D\right)$ may require a great amount of time if |U| is large. Therefore, a natural idea of accelerating Algorithm 1 is that accelerating the computation of $SIG_{\lambda }\left(a_{i},\;B,\;D\right)$ according to computational properties of the λ-conditional entropy.

## 3. Accelerated Computation of λ-Conditional Entropy

In the following, we concentrate on the computational characteristic of λ-conditional entropy. Firstly, we review the following theorem in [44].

**Theorem** **1.** *Let (U,A) be a fuzzy information system with a fuzzy relation $R_{B}$ for each B⊆A. For any fuzzy set X∈F(U),*

(6) $$\underline{R_{B}}X\left(x_{i}\right)=\sup\left\{\lambda :{\left[x_{i}\right]}_{B}^{\lambda }\subseteq X\right\}.$$

Here, ${\left[x_{i}\right]}_{B}^{\lambda }$ with $\lambda \leq \underline{R_{B}}X\left(x_{i}\right)$ is a basic fuzzy granule with respect to *B* to characterize the inner structure of *X*. Let *X* be ${\left[x_{i}\right]}_{D}$. Then, ${\left[x_{i}\right]}_{B}^{\lambda_{i}}$ with $\lambda_{i}=\underline{R_{B}}{\left[x_{i}\right]}_{D}\left(x_{i}\right)$ is the biggest granule contained in ${\left[x_{i}\right]}_{D}$.

Let (U,A∪D) be a fuzzy decision system with $U=\{x_{1},\;x_{2},\;\ldots ,\;x_{n}\}$ and B⊆A. Denote

(7) $$U_{B}^{*}=\left\{x_{i}:\left|{\left[x_{i}\right]}_{B}^{\lambda_{i}}\cap {\left[x_{i}\right]}_{D}\right|<\left|{\left[x_{i}\right]}_{B}^{\lambda_{i}}\right|,\;x_{i}\in U\right\}$$

as the object set in which each object $x_{i}$ satisfies $\left|{\left[x_{i}\right]}_{B}^{\lambda_{i}}\cap {\left[x_{i}\right]}_{D}\right|<\left|{\left[x_{i}\right]}_{B}^{\lambda_{i}}\right|$. It is obvious to have $U_{B}^{*}\subseteq U$. We then have the following property.

**Property** **1.** *Let (U,A∪D) be a fuzzy decision system with $U=\{x_{1},\;x_{2},\;\ldots ,\;x_{n}\}$ and B⊆A. Then*

(8) $$H_{\lambda }\left(D|B\cup \left\{a\right\}\right)=-\frac{1}{\left|U\right|}\sum_{x_{i}\in U_{B}^{*}}\left(\left|{\left[x_{i}\right]}_{B\cup \{a\}}^{\lambda_{i}}\cap {\left[x_{i}\right]}_{D}\right|\log\frac{\left|{\left[x_{i}\right]}_{B\cup \{a\}}^{\lambda_{i}}\cap {\left[x_{i}\right]}_{D}\right|}{\left|{\left[x_{i}\right]}_{B\cup \{a\}}^{\lambda_{i}}\right|}\right)$$

*holds for any a∈A∖B.*

**Proof.**  Assume that $U\setminus U_{B}^{*}\neq \emptyset$. Then, for any $x_{i}\in U\setminus U_{B}^{*}$, we have $\left|{\left[x_{i}\right]}_{B}^{\lambda_{i_{0}}}\cap {\left[x_{i}\right]}_{D}\right|=\left|{\left[x_{i}\right]}_{B}^{\lambda_{i}}\right|$, which is equivalent to ${\left[x_{i}\right]}_{B}^{\lambda_{i}}\subseteq {\left[x_{i}\right]}_{D}$. Then, according to Theorem 1, it is obtained that $\underline{R_{B}}{\left[x_{i}\right]}_{D}\left(x_{i}\right)\geq \lambda_{i}$. Because of $\underline{R_{B}}{\left[x_{i}\right]}_{D}\left(x_{i}\right)\leq \underline{R_{A}}{\left[x_{i}\right]}_{D}\left(x_{i}\right)=\lambda_{i}$, we have $\underline{R_{B}}{\left[x_{i}\right]}_{D}\left(x_{i}\right)=\lambda_{i}$, which yields $\underline{R_{B\cup \{a\}}}{\left[x_{i}\right]}_{D}\left(x_{i}\right)=\lambda_{i}$ and then ${\left[x_{i}\right]}_{B\cup \{a\}}^{\lambda_{i}}\subseteq {\left[x_{i}\right]}_{D}$ for any a∈A∖B. Therefore, $\left|{\left[x_{i}\right]}_{B\cup \{a\}}^{\lambda_{i}}\cap {\left[x_{i}\right]}_{D}\right|=\left|{\left[x_{i}\right]}_{B\cup \{a\}}^{\lambda_{i}}\right|$ and then

$$-\frac{1}{\left|U\right|}\left|{\left[x_{i}\right]}_{B\cup \{a\}}^{\lambda_{i}}\cap {\left[x_{i}\right]}_{D}\right|\log\frac{\left|{\left[x_{i}\right]}_{B\cup \{a\}}^{\lambda_{i}}\cap {\left[x_{i}\right]}_{D}\right|}{\left|{\left[x_{i}\right]}_{B\cup \{a\}}^{\lambda_{i}}\right|}=0,$$

which yields

$$\begin{array}{cc} H_{\lambda }\left(D|B\cup \left\{a\right\}\right) & =-\frac{1}{\left|U\right|}\sum_{x_{i}\in U}\left(\left|{\left[x_{i}\right]}_{B\cup \{a\}}^{\lambda_{i}}\cap {\left[x_{i}\right]}_{D}\right|\log\frac{\left|{\left[x_{i}\right]}_{B\cup \{a\}}^{\lambda_{i}}\cap {\left[x_{i}\right]}_{D}\right|}{\left|{\left[x_{i}\right]}_{B\cup \{a\}}^{\lambda_{i}}\right|}\right)\\ & =-\frac{1}{\left|U\right|}\sum_{x_{i}\in U_{B}^{*}}\left(\left|{\left[x_{i}\right]}_{B\cup \{a\}}^{\lambda_{i}}\cap {\left[x_{i}\right]}_{D}\right|\log\frac{\left|{\left[x_{i}\right]}_{B\cup \{a\}}^{\lambda_{i}}\cap {\left[x_{i}\right]}_{D}\right|}{\left|{\left[x_{i}\right]}_{B\cup \{a\}}^{\lambda_{i}}\right|}\right)\\ & \;\;\;\;\;-\frac{1}{\left|U\right|}\sum_{x_{i}\in U\setminus U_{B}^{*}}\left(\left|{\left[x_{i}\right]}_{B\cup \{a\}}^{\lambda_{i}}\cap {\left[x_{i}\right]}_{D}\right|\log\frac{\left|{\left[x_{i}\right]}_{B\cup \{a\}}^{\lambda_{i}}\cap {\left[x_{i}\right]}_{D}\right|}{\left|{\left[x_{i}\right]}_{B\cup \{a\}}^{\lambda_{i}}\right|}\right)\\ & =-\frac{1}{\left|U\right|}\sum_{x_{i}\in U_{B}^{*}}\left(\left|{\left[x_{i}\right]}_{B\cup \{a\}}^{\lambda_{i}}\cap {\left[x_{i}\right]}_{D}\right|\log\frac{\left|{\left[x_{i}\right]}_{B\cup \{a\}}^{\lambda_{i}}\cap {\left[x_{i}\right]}_{D}\right|}{\left|{\left[x_{i}\right]}_{B\cup \{a\}}^{\lambda_{i}}\right|}\right) \end{array}.$$

☐

Assume that the similarity relation $R_{B}\left(x_{i},x_{j}\right)$ has been computed for any $x_{i}\in U$ and $x_{j}\in U$. Then, according to Property 1, the time complexity of $H_{\lambda }\left(D|B\cup \left\{a\right\}\right)$ is $O(|U_{B}^{*}||U|)$, which is generally less than $O(|U{|}^{2})$ since $U_{B}^{*}\subseteq U$ holds.

Denote

(9) $$U_{B}^{x_{i}}=\left\{x_{j}:{\left[x_{i}\right]}_{B}^{\lambda_{i}}\left(x_{j}\right)=\lambda_{i},x_{j}\in U\right\}$$

as the object set in which each object belongs to the fuzzy set ${\left[x_{i}\right]}_{B}^{\lambda_{i}}$ with the degree being $\lambda_{i}$. Since

$${\left[x_{i}\right]}_{B}^{\lambda_{i}}\left(x_{j}\right)=\left\{\begin{array}{c} \lambda_{i},\;1-R_{B}\left(x_{i},x_{j}\right)<\lambda_{i};\\ 0,\;\mathrm{otherwise}, \end{array}\right.$$

then, for any $x_{j}\in U\setminus U_{B}^{x_{i}}$, it is easily obtained that ${\left[x_{i}\right]}_{B}^{\lambda_{i}}\left(x_{j}\right)=0$. Furthermore, we have the following property.

**Property** **2.** *Let (U,A∪D) be a fuzzy decision system with $U=\{x_{1},\;x_{2},\;\ldots ,\;x_{n}\}$ and B⊆A. Then, for any a∈A∖B, we have*

(10) $$\left|{\left[x_{i}\right]}_{B\cup \{a\}}^{\lambda_{i}}\right|=\sum_{x_{j}\in U_{B}^{x_{i}}}{\left[x_{i}\right]}_{B\cup \{a\}}^{\lambda_{i}}\left(x_{j}\right)$$

*and*

(11) $$\left|{\left[x_{i}\right]}_{B\cup \{a\}}^{\lambda_{i}}\cap {\left[x_{i}\right]}_{D}\right|=\sum_{x_{j}\in \left(U_{B}^{x_{i}}\cap {\left[x_{i}\right]}_{D}\right)}{\left[x_{i}\right]}_{B\cup \{a\}}^{\lambda_{i}}\left(x_{j}\right).$$

**Proof.**  Assume that $U\setminus U_{B}^{x_{i}}\neq \emptyset$. Then, for any a∈A∖B and any $x_{j}\in U\setminus U_{B}^{x_{i}}$, it is obtained that the fuzzy similarity relation $R_{B\cup \{a\}}=R_{B}\cap R_{\left\{a\right\}}\subseteq R_{B}$ and $1-R_{B}\left(x_{i},x_{j}\right)\geq \lambda_{i}$, which yields $1-R_{B\cup \{a\}}\left(x_{i},x_{j}\right)\geq 1-R_{B}\left(x_{i},x_{j}\right)\geq \lambda_{i}$ and then ${\left[x_{i}\right]}_{B\cup \{a\}}^{\lambda_{i}}\left(x_{j}\right)=0$. Therefore, we have

$$\begin{array}{cc} \left|{\left[x_{i}\right]}_{B\cup \{a\}}^{\lambda_{i}}\right| & =\sum_{x_{j}\in U}{\left[x_{i}\right]}_{B\cup \{a\}}^{\lambda_{i}}\left(x_{j}\right)\\ & =\sum_{x_{j}\in U_{B}^{x_{i}}}{\left[x_{i}\right]}_{B\cup \{a\}}^{\lambda_{i}}\left(x_{j}\right)+\sum_{x_{j}\in U\setminus U_{B}^{x_{i}}}{\left[x_{i}\right]}_{B\cup \{a\}}^{\lambda_{i}}\left(x_{j}\right)\\ & =\sum_{x_{j}\in U_{B}^{x_{i}}}{\left[x_{i}\right]}_{B\cup \{a\}}^{\lambda_{i}}\left(x_{j}\right) \end{array}$$

and

$$\begin{array}{cc} \left|{\left[x_{i}\right]}_{B\cup \{a\}}^{\lambda_{i}}\cap {\left[x_{i}\right]}_{D}\right| & =\sum_{x_{j}\in {\left[x_{i}\right]}_{D}}{\left[x_{i}\right]}_{B\cup \{a\}}^{\lambda_{i}}\left(x_{j}\right)\\ & =\sum_{x_{j}\in \left(U_{B}^{x_{i}}\cap {\left[x_{i}\right]}_{D}\right)}{\left[x_{i}\right]}_{B\cup \{a\}}^{\lambda_{i}}\left(x_{j}\right)+\sum_{x_{j}\in \left(\left(U\setminus U_{B}^{x_{i}}\right)\cap {\left[x_{i}\right]}_{D}\right)}{\left[x_{i}\right]}_{B\cup \{a\}}^{\lambda_{i}}\left(x_{j}\right)\\ & =\sum_{x_{j}\in \left(U_{B}^{x_{i}}\cap {\left[x_{i}\right]}_{D}\right)}{\left[x_{i}\right]}_{B\cup \{a\}}^{\lambda_{i}}\left(x_{j}\right). \end{array}$$

☐

Substituting Equations (10) and (11) into Equation (8), we then have

(12) $$H_{\lambda }\left(D|B\cup \left\{a\right\}\right)=-\frac{1}{\left|U\right|}\sum_{x_{i}\in U_{B}^{*}}\left(\left(\sum_{x_{j}\in \left(U_{B}^{x_{i}}\cap {\left[x_{i}\right]}_{D}\right)}{\left[x_{i}\right]}_{B\cup \{a\}}^{\lambda_{i}}\left(x_{j}\right)\right)\log\frac{\left(\sum_{x_{j}\in \left(U_{B}^{x_{i}}\cap {\left[x_{i}\right]}_{D}\right)}{\left[x_{i}\right]}_{B\cup \{a\}}^{\lambda_{i}}\left(x_{j}\right)\right)}{\left(\sum_{x_{j}\in U_{B}^{x_{i}}}{\left[x_{i}\right]}_{B\cup \{a\}}^{\lambda_{i}}\left(x_{j}\right)\right)}\right).$$

**Corollary** **1.** *Let (U,A∪D) be a fuzzy decision system with $U=\{x_{1},\;x_{2},\;\ldots ,\;x_{n}\}$ and B⊆A. Then, for any a∈A∖B, we have*

(13) $$U_{B\cup \{a\}}^{x_{i}}\subseteq U_{B}^{x_{i}}.$$

**Proof.**  For any $x_{j}\in U\setminus U_{B}^{x_{i}}$, we have ${\left[x_{i}\right]}_{B}^{\lambda_{i}}\left(x_{j}\right)=0$. It can be obtained from the proof process of Property 2 that ${\left[x_{i}\right]}_{B\cup \{a\}}^{\lambda_{i}}\left(x_{j}\right)=0$ holds for any a∈A∖B, which yields $x_{j}\in U\setminus U_{B\cup \{a\}}^{x_{i}}$. Thus, $\left(U\setminus U_{B}^{x_{i}}\right)\subseteq \left(U\setminus U_{B\cup \{a\}}^{x_{i}}\right)$. which implies $U_{B\cup \{a\}}^{x_{i}}\subseteq U_{B}^{x_{i}}$. ☐

Assume that the similarity relation $R_{B}\left(x_{i},x_{j}\right)$ has been computed for any $x_{i}\in U$ and $x_{j}\in U$. Then, according to Equation (12), the time complexity of $H_{\lambda }\left(D|B\cup \left\{a\right\}\right)$ is $O(C|U_{B}^{*}|)$, which is generally less than $O(|U{|}^{2})$ since both C≤|U| and $|U_{B}^{*}\left|\leq |U\right|$ hold. Here, $C=\max\{|U_{B}^{x_{i}}|:x_{i}\in U_{B}^{*}\}$. Therefore, according to Properties 1 and 2, we can use Equation (12) to compute $H_{\lambda }\left(D|B\cup \left\{a\right\}\right)$ and then obtain an accelerated algorithm in the following.

Compared with Algorithm 1, there exist three aspects of differences in Algorithm 2. First, Algorithm 2 needs to set $U_{B}^{*}$ and $U_{B}^{x_{i}}$ ($x_{i}\in U$) to *U* in Steps 1–4. Second, the evaluation measure $H_{\lambda }\left(D|B\cup \left\{a_{i}\right\}\right)$ is improved to compute according to Equation (12) in Step 10, in which $U_{B\cup \{a_{i}\}}^{*}$ can be automatically acquired without additional computation. Here, the complexity of computing $H_{\lambda }\left(D|B\cup \left\{a_{i}\right\}\right)$ is $O(C|U_{B}^{*}|)$, where $C=\max\{|U_{B}^{x_{i}}|:x_{i}\in U_{B}^{*}\}$. Third, $U_{B}^{*}$ and $U_{B}^{x_{i}}$ ($x_{i}\in U$) are iteratively updated in Steps 16–20, and Steps 17–20 need $O(C|U_{B}^{*}|)$. Furthermore, the main procedure of Algorithm 2 for selecting features, namely Steps 8–22, needs to be run at most |A| times, so the time complexity is $O(C|U_{B}^{*}||A{|}^{2})$. However, the main process Steps 5–14 in Algorithm 1 for selecting features requires $O(|U{|}^{2}|A{|}^{2})$. It should be pointed out that both $|U_{B}^{*}|$ and *C* may monotonously decrease in the iteration process of Algorithm 2, which mainly contributes to accelerate computation.

**Algorithm 2:** Accelerating computation of an ε-approximate reduct of a fuzzy decision system.

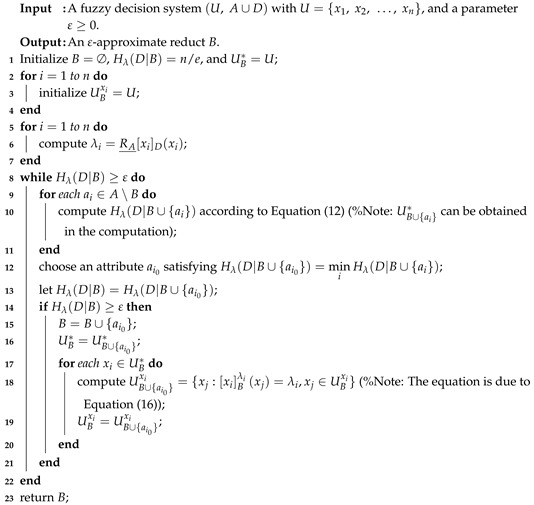



## 4. Numerical Experiment

In this section, numerical experiments are conducted to assess the performance of Algorithm 2. The experiment mainly focuses on showing the computational efficiency of Algorithm 2. In order to achieve the task, nine data sets are downloaded from UCI Repository of machine learning databases. The data sets are briefly described in Table 1.

### 4.1. Pretreatment of the Data Sets and Design of the Experiment

For each data set, the object set, conditional attribute set and decision attribute set are denoted by *U*, *A*, and *D*, respectively. If there are some real-valued conditional attributes in *A*, then, for each real-valued attribute a∈A, the attribute value of each object is normalized according to the method in [44] as

(14) $$\tilde{a}\left(x_{i}\right)=\frac{a\left(x_{i}\right)-\min_{j}a\left(x_{j}\right)}{\max_{j}a\left(x_{j}\right)-\min_{j}a\left(x_{j}\right)},\;x_{i}\in U,$$

so that $\tilde{a}\left(x_{i}\right)\in \left[0,1\right]$ for each $x_{i}\in U$. Here, *a* is still used to denote the corresponding normalized conditional attribute for notational simplicity.

The experiment was designed as follows. Given one of the pretreated data sets, the objects were randomly divided into 20 approximately equal parts. The first part was taken as the 1st data set, the combination of both the first and the second parts was regarded as the 2nd data set, the combination of the anterior three parts was regarded as the 3rd data set, ···, and the combination of all twenty parts was taken as the 20th data set. For each of the generated 20 data sets, a fuzzy relation for each normalized conditional attribute *a* is defined as

(15) $$R_{\left\{a\right\}}\left(x_{i},x_{j}\right)=1-\left|a\left(x_{i}\right)-a\left(x_{j}\right)\right|,\;x_{i},x_{j}\in U_{k}.$$

On the other hand, a special fuzzy relation, namely an equivalence relation, is defined for each nominal attribute a∈A by

(16) $$R_{\left\{a\right\}}\left(x_{i},x_{j}\right)=\left\{\begin{array}{c} 1,\;a\left(x_{i}\right)=a\left(x_{j}\right);\\ 0,\;\mathrm{otherwise} \end{array},\right.$$

where $x_{i},x_{j}\in U_{k}$. Here, $U_{k}$ is the universe determined by the *k*-th data set. In this way, a fuzzy decision system $\left(U_{k},\;A\cup D\right)$ is formed for the *k*-th data set. Then, Algorithms 1 and 2 were used to obtain the computation time of these fuzzy decision systems, respectively. Furthermore, the “ten-fold approach” was also used to access the efficiency of the fast algorithm proposed in this paper. Specifically, for each of the pretreated data sets, the instances were randomly divided into 10 approximately equal parts. The *k*-th part was removed and the remainder was taken as the *k*-th data set, which generates the ten data sets called the ten-fold data sets. Then, the fuzzy relations for real-valued attributes and nominal attributes were defined according to Equations (18) and (19), respectively, which then formed a fuzzy decision system for each of the ten-fold data sets. Algorithms 1 and 2 were used to obtain the computation time of the fuzzy decision systems, respectively. Moreover, it should be pointed out that the output results obtained by both Algorithms 1 and 2 are the same for the same threshold values ε. The parameter ε determines the number of the selected features. The smaller the threshold value ε is, the more selected features there are and thus the more computation time is needed. Therefore, the parameter ε in both Algorithms 1 and 2 was set to 0. The experiment was performed by MATLAB R2016a on a personal computer with Intel(R) Core(TM) i7-4510U CPU @2.00 GHz configuration, 8 G Memory, and the 64-bit Windows 7 system.

### 4.2. Comparison of Computation Time of Algorithms 1 and 2

#### 4.2.1. Comparison of Computation Time on 20 Data Sets Generated by Each Data Set

**Figure 1:**
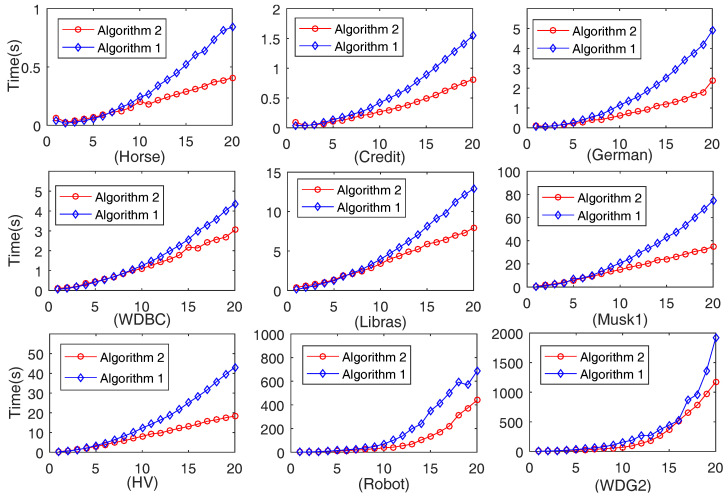
Computation time of Algorithms 1 and 2 with the increase of the size of each data set.

The computation time on 20 data sets generated by each data set respectively obtained by Algorithms 1 and 2 is depicted in Figure 1. For each of the sub-figures in Figure 1, the x-coordinate indicates the generated data sets and the number *k* expresses the *k*-th data set. In other words, the x-coordinate expresses the size of each data set and the number *k* is factually (5∗k)% data of original data sets. On the other hand, the y-coordinate shows the running time (in seconds).

It is seen from Figure 1 that, for each data set, with the increase in data size, both Algorithms 1 and 2 require more time. At the beginning, the two algorithms cost an almost equivalent amount of time. Algorithm 2 needs a little more time relative to Algorithm 1 since the advantage of Algorithm 2 is limited by a smaller data set size. Algorithm 2 may need more time to run Steps 17–20. However, with the increase in data set size, Algorithm 2 obviously requires less running time than Algorithm 1. Therefore, the proposed Algorithm 2 is efficient and can be regarded as an accelerated version of Algorithm 1.

#### 4.2.2. Comparison of Computation Time on Ten-Folds Data Sets Produced by Each Data Set

**Figure 2:**
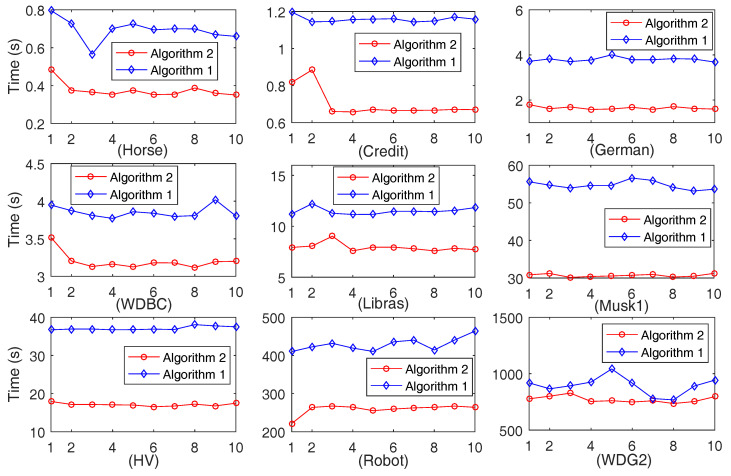
Computation time of Algorithms 1 and 2 on ten-fold data sets generated by each data set.

**Figure 3:**
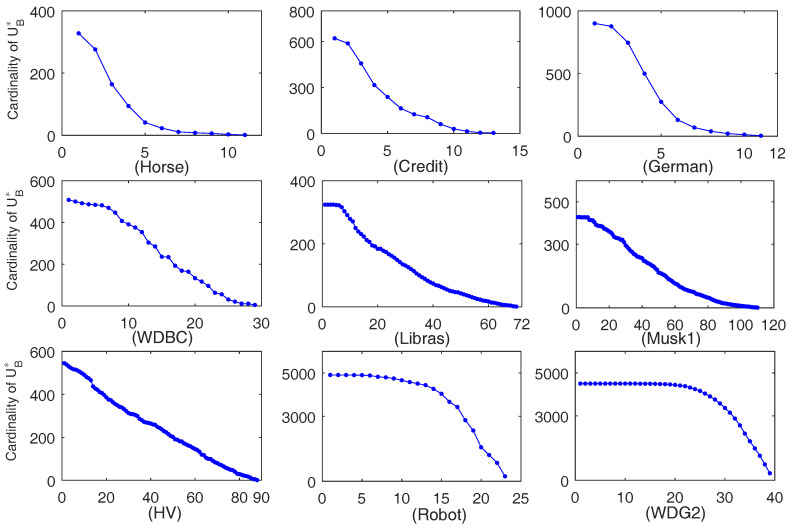
Variation of $|U_{B}^{*}|$ with the increase of iteration number in Algorithm 2.

The computation time of ten-fold data sets generated by each data set is depicted in Figure 2. For each of the sub-figures in Figure 2, the x-coordinate indicates the generated data sets and the number *i* expresses the *i*-th data set, and the y-coordinate shows the running time (in second). Furthermore, the average computation time is listed in Table 2. In addition, the average cardinalities of the selected feature subset, which is expressed by |·|, are also listed in the 3rd and 5th columns of Table 2. Moreover, in order to illustrate the variation tendency of $|U_{B}^{*}|$ in the iteration process of the proposed Algorithm 2, the relevant result obtained by one of the ten-fold data sets is depicted in Figure 3. For each of the sub-figures in Figure 3, the x-coordinate indicates the number of iterations in Algorithm 2 and the y-coordinate expresses the cardinality of $U_{B}^{*}$.

It can be clearly seen in Figure 2 and Table 2 that, for each of the data sets, Algorithm 2 requires less time than Algorithm 1 for the ten-fold data sets. Especially for data sets German, Musk1, HV, and Robot, Algorithm 2 requires much less time and needs approximately no greater than 60% of the running time of Algorithm 1. Thus, it seems that Algorithm 2 requires significantly less running time for the data sets with a larger size or with more features. Moreover, the results of the 3rd and the 5th columns in Table 2 verify that the selected features respectively obtained by Algorithms 1 and 2 are the same. In addition, it can be seen from Figure 3 that $|U_{B}^{*}|$ does monotonously decrease with the increase of the iteration number. In fact, the decrease of $|U_{B}^{*}|$ contributes to the accelerating computation of Algorithm 2. Therefore, Algorithm 2 is validated to be effective again on the ten-fold data sets.

## 5. Conclusions

Based on the existing feature selection algorithm, by utilizing a fuzzy rough set-based information entropy, an accelerated feature selection algorithm according to the computational properties of fuzzy rough set-based information entropy, in which the entropy is computed by a lower time complexity, is presented in this paper. The numerical experiment results demonstrate that the algorithm can effectively decrease computation time and thus is efficient and effective. In future work, the proposed fast feature selection algorithm will be considered to deal with a dynamic data environment in which new instances or new features are added. 

## Figures and Tables

**Table 1:** Description of the data sets.

Data Set	Abbreviation of Data Set	Number of Objects	Number of Conditional Attributes			Number of Classes
			All	Nominal	Real-Valued	
Horse Colic	Horse	368	22	15	7	2
Credit Approval	Credit	690	15	9	6	2
German Credit Data	German	1000	20	13	7	2
Wisconsin Diagnostic Breast Cancer	WDBC	569	30	0	30	2
Libras Movement	Libras	360	90	0	90	15
Musk (Version 1)	Musk1	476	166	0	166	2
Hill-Valley	HV	606	100	0	100	2
Wall-Following Robot Navigation Data	Robot	5456	24	0	24	4
Waveform Database Generator (Version 2)	WDG2	5000	40	0	40	3

**Table 2:** Average results of Algorithms 1 and 2 obtained from the ten-fold data sets.

Data Set	Algorithm 2			Algorithm 1 [44]	
	Average Running Time (s)	|·|		Average Running Time (s)	|·|
Horse	0.38	12.7		0.69	12.7
Credit	0.70	13.9		1.16	13.9
German	1.65	12.9		3.79	12.9
WDBC	3.20	30.0		3.85	30.0
Libras	7.94	71.4		11.48	71.4
Musk1	30.69	112.4		54.69	112.4
HV	17.12	90.0		37.11	90.0
Robot	259.00	24.0		428.85	24.0
WDG2	771.46	40.0		894.15	40.0
